# Genomic insights into an endophytic *Streptomyces* sp. VITGV156 for antimicrobial compounds

**DOI:** 10.3389/fmicb.2024.1407289

**Published:** 2024-06-03

**Authors:** Pattapulavar Veilumuthu, T. Nagarajan, Sharayu Magar, Sasikumar Sundaresan, Lenus Joy Moses, Thomas Theodore, John Godwin Christopher

**Affiliations:** ^1^Department of Biomedical Sciences, School of BioSciences and Technology, Vellore Institute of Technology, Vellore, India; ^2^Department of Biological Sciences, SRM University-AP, Amaravathi, India; ^3^Department of Biochemistry, School of Biological Sciences, Madurai Kamaraj University, Madurai, India; ^4^School of Chemical Engineering, Vellore Institute of Technology, Vellore, India

**Keywords:** *Streptomyces* sp. VITGV156, endophytes, tomato plant, kendomycin, MRSA, antiSMASH, biosynthetic gene cluster

## Abstract

Endophytic *Streptomyces* sp. are recognized as a potential resource for valuable natural products but are less explored. This study focused on exploring endophytic *Streptomyces* species residing within tomato plants (*Solanum lycopersicum*) harboring genes for the production of a novel class of antibiotics. Our research involved the isolation and characterization of *Streptomyces* sp. VITGV156, a newly identified endophytic *Streptomyces* species that produces antimicrobial products. VITGV156 harbors a genome of 8.18 mb and codes 6,512 proteins, of which 4,993 are of known function (76.67%) and 1,519 are of unknown function (23.32%). By employing genomic analysis, we elucidate the genome landscape of this microbial strain and shed light on various BGCs responsible for producing polyketide antimicrobial compounds, with particular emphasis on the antibiotic kendomycin. We extended our study by evaluating the antibacterial properties of kendomycin. Overall, this study provides valuable insights into the genome of endophytic *Streptomyces* species, particularly *Streptomyces* sp. VITGV156, which are prolific producers of antimicrobial agents. These findings hold promise for further research and exploitation of pharmaceutical compounds, offering opportunities for the development of novel antimicrobial drugs.

## Introduction

Antibiotics are a cornerstone of modern medicine and have become inevitable in healthcare. However, the global emergence of antimicrobial resistance (AMR) has restricted the use of many antibiotics and initiated the quest for the discovery of novel antibiotic compounds (Antimicrobial Resistance Collaborators et al., 2022; Laxminarayan, 2022). Several plant and microbial sources have been screened for the presence of bioactive metabolites, and microbial sources have gained increasing attention owing to their resourcefulness and ease of production (Tanaka and Omura, 1990; Kekuda et al., 2015). Among the microbial sources, *Actinobacteria* stands atop in the discovery of antimicrobial compounds, and *Streptomyces* is the most prominent. *Streptomyces* is a large class of Gram-positive bacteria well recognized for its ability to produce antimicrobial compounds.

As of July 12, 2023, the *Streptomyces* genus encompasses 1,179 species and 73 subspecies distributed across three genera. Among these, 590 *Streptomyces* genomes have been sequenced, supported by 4,903 SRA data entries in the NCBI database. More than 45% of the identified antimicrobial compounds are attributed to several species of *Streptomyces* (Kinkel et al., 2014; Law et al., 2017). In addition, they are prolific producers of bioactive compounds with antifungal, antiviral, antioxidant, and anticancer properties (Loganathan et al., 2013; Karthik et al., 2014; Ayswaria et al., 2020; Alam et al., 2022; Lacey and Rutledge, 2022). Therefore, the isolation and characterization of *Streptomyces* sp. from diverse ecosystems and niches have been adopted worldwide for bioprospecting (Chhetri et al., 2024). Screening of *Streptomyces* sp. from previously unexplored areas not only paves the way for the isolation of novel species but also provides interesting bioactive compounds (Law et al., 2019).

*Streptomyces* spp. are classified as saprophytes that help in the recycling of dead and decaying organic matter in soil. Thus, soil has become the major habitat for all *Streptomyces* species. However, several species have been reported to exhibit endophytic relationships with plant hosts (Seipke et al., 2012; Olanrewaju and Babalola, 2019). They enter the plant host through natural openings, root colonization, association with other endophytic organisms, and seed transmission and colonize the roots, stem, and leaves of the host plant (Coombs and Franco, 2003; Chen et al., 2016; Kandel et al., 2017; Khare et al., 2018). The endophytic association of *Streptomyces* with plant hosts benefits them in many ways, including conferring resistance to infection and plant growth promotion (Quecine et al., 2008; Verma et al., 2011; Vurukonda et al., 2018; Worsley et al., 2020). In turn, endophytic relationships also have a significant impact on *Streptomyces*. Several studies have shown that endophytic relationships promote the evolution of secondary metabolic pathways (Ayswaria et al., 2020; Al-Quwaie, 2024) in *Streptomyces*. This results in the production of novel secondary metabolites that have ecological and biotechnological implications. Emerging advances in genome mining techniques have further revealed the presence of latent BGCs in *Streptomyces* genomes, underscoring their potential for novel drug discovery.

Polyketide antibiotics, derived from *Streptomyces* bacteria, represent a vital class of antimicrobial compounds. Many known antibiotics, including renowned examples such as erythromycin, tetracycline, and streptomycin, are polyketide antibiotics produced by various *Streptomyces* sp. strains. These antibiotics are synthesized through polyketide synthase enzymes, which assemble diverse chemical structures. These antibiotics exhibit broad-spectrum activity against various bacterial pathogens, making them indispensable in clinical medicine and agriculture. Kendomycin, a macrocyclic polyketide, is produced by various *Streptomyces* species, including *S. violaceoruber* (Yuan et al., 2004). Recent studies have detailed multiple synthesis methods for kendomycin, underscoring its importance in biological investigations. This compound has cytotoxic effects on various human tumor cell lines, such as MCF-7, HMO2, and HepG2. Moreover, previous findings indicate that kendomycin disrupts mammalian proteasome functions and triggers apoptosis in U937 cells. Its potent antibacterial activity extends to both Gram-negative and Gram-positive bacteria, including methicillin-resistant *Staphylococcus aureus* (MRSA) (Bode and Zeeck, 2000; Elnakady et al., 2007; Duggal et al., 2011; Hesje et al., 2011; Tattevin, 2011). The identification of novel *Streptomyces* spp. capable of producing a new class of antibiotics is valuable for both exploring the biosynthetic pathways and commercial production of antimicrobial compounds.

Tomatoes stand out for their exceptional nutritional value, boasting an array of bioactive compounds, including carotenoids, polyphenols, ascorbate, and folate. This nutritional richness positions them as paramount agricultural products, providing vital nutrients. Over its lifecycle, the tomato plant interacts with diverse microorganisms. Notably, endophytic actinobacteria find sanctuaries within plants, actively contributing to plant defense mechanisms by synthesizing pharmaceutical compounds (Kiran et al., 2022). Endophytic *Streptomyces* colonizing tomato plants act as biocontrol agents and enhance growth in the host plant (Goudjal et al., 2016; Passari et al., 2019; Ling et al., 2020; Devi et al., 2022). Among many biocontrol agents, kendomycin is a small natural molecule that has gained significant attention due to its strong antimicrobial activity.

Despite the beneficial effects of endophytic *Streptomyces* and the metabolites they produce, their BGCs remain poorly understood. Therefore, this study aimed to isolate and characterize endophytic *Streptomyces* strains capable of producing novel antimicrobial compounds from tomato plants from the perspective of the genome landscape. In this study, a *Streptomyces* species (VITGV156) was isolated and shown to have strong antimicrobial activity against *Escherichia coli*. The whole-genome sequence was determined, and the taxonomic identity was determined. Mining of the VITGV156 genome revealed the presence of BGCs for several polyketide antibiotics. The antimicrobial activity of a polyketide antibiotic was also analyzed.

## Materials and methods

### Chemicals and media

The chemicals, media components, and standard antibiotics used in the study were purchased from HiMedia Pvt. Ltd., Mumbai, India and Sigma-Aldrich Corporation, Bangalore, India. All solvents used were of analytical/HPLC grade and were purchased from SD Fine Chem Limited, India. The antibiotics and fine chemicals used were purchased from SD Fine Chem Limited, India. The antibiotic kendomycin (BIA-K1143) was purchased from BioAustralis Fine Chemicals, Australia.

### Sample collection

Healthy tomato plants (cv. Arka Rakshak) were collected from tomato field fields in and around Madurai, India. The aerial regions of 10 plants were carefully removed and washed with tap water. The samples were placed in airtight bags, taken to the laboratory, and processed within 4–6 h.

### Isolation of endophytic *Streptomyces* sp. from tomato plants

The collected plant samples were processed aseptically in Biological Safety Cabinet Class II. The samples were sterilized by sequential immersion in 70% (v/v) ethanol for 5 min and sodium hypochlorite solution (0.9%, w/v, available chlorine) for 20 min. The surface-sterilized root samples were washed in sterile water three times to remove surface sterilization agents. Next, the root samples were soaked in 10% (w/v) NaHCO3 solution for 10 min to retard the growth of endophytic fungi. The sample was divided into small fragments (0.5 cm × 0.5 cm) and placed on IS2 media (0.4% yeast extract powder, 1% malt extract powder, 0.4% dextrose, and 2% agar). Fifteen ppm (w/v) nalidixic acid was added to suppress the growth of contaminating bacteria, and the mixture was then incubated at room temperature for 15 days at 30°C. The colonies that emerged were subsequently purified by further streaking on ISP2 plates two times. A pure culture was maintained in glycerol (20%, v/v) at −80°C.

### Identification and characterization of *Streptomyces* sp. VITGV156

The strain *Streptomyces* sp. VITGV156 was identified via phenotypic and genotypic characterization. The morphology of the strain was examined using both phase contrast microscopy (MT5210/MEIJI) and scanning electron microscopy (Carl Zeiss-Evo 18). The preparation of the samples for scanning electron microscopy was performed according to Chanama et al. (2023) and Veilumuthu and Christopher (2022a). These imaging techniques seamlessly unveiled the intricate morphological attributes of the strain, rendering a vivid depiction of its distinctive characteristics.

### Genome annotation and comparative genomic analysis

Gene prediction and functional annotation were performed by Rapid Annotation using Subsystem Technology (RAST) (Brettin et al., 2015). This annotation was further complemented with the COG (Cluster of Orthologous Groups of Proteins) (Tatusov et al., 2000) database^1^ with an *E*-value cutoff of 1e−02 to gain insights into biological, molecular, and cellular function-encoding genes. For the analysis of 16S rRNA sequences within the *Streptomyces* genus, 16S rRNA sequences were retrieved from NCBI. Alignment and model selection were performed using MUSCLE v3.8.1551 (Edgar, 2004) and MEGAX v11.0.13 (Tamura et al., 2021), respectively. Maximum likelihood phylogenies were generated using RAxML v8.2.12 (Stamatakis, 2014) with the GTRGAMMA (mtREV24 + G + I + F) nucleotide substitution model and a bootstrap value of 100. The resulting phylogenetic tree was visualized using the iTOL platform (Letunic and Bork, 2021). The average nucleotide identity scores for the selected genomes were calculated using pyani-0.2.10. GGDC 3.0 was used for genome-to-genome comparisons. A circular map of *Streptomyces* sp. VITGV156 was generated using the Proksee tool (Grant et al., 2023). Orthology was determined within protein datasets from 10 genomes using the Reciprocal Best Hits (RBH) BLAST method implemented in ProteinOrtho (Klemm et al., 2023), with an E-value threshold set at 1e−5, along with default parameters. The biosynthetic clusters involved in the synthesis of secondary metabolites were analyzed using antiSMASH 7.0 (Blin et al., 2023).

### Biomass production and metabolite extraction

The *Streptomyces* strain VITGV156 is an endophyte in tomato plants that is cultured in ISP2 broth. Once the culture reached the lag phase, it was divided equally into 10 500-ml culture flasks, each containing 300 mL of ISP2 medium (Veilumuthu et al., 2023). These culture flasks were kept in a laboratory shaking incubator at 150 rpm for 21 days at 30°C. Following incubation, the cells were removed by centrifugation (2,000 rpm, 5 min, 4°C), and the supernatant was further filtered through Whatman paper to obtain a cell-free filtrate. The secondary metabolite was recovered from the broth using a two-phase solvent extraction system with ethyl acetate (1:1). Then this mixture was vigorously shaken for 10 min and kept in a shaker for 24 h at 200 rpm. This was allowed to settle for 30 min. Organic phases were collected and concentrated in a rotary evaporator (model RE100-Pro) at 54°C and 80 rpm (Veilumuthu et al., 2022).

### Chemical analysis of LC-HRMS

The ethyl acetate extract derived from the culture filtrate of *Streptomyces* sp. VITGV156 was subjected to LC-HRMS analysis. Data analysis was conducted using the LC-Solution tools provided by Shimadzu Corporation, Kyoto, Japan. A Waters^®^ Micromass^®^ Q-Tof micro^™^ mass spectrometer was used for the LC-HRMS-ESI.

### Bacterial strains and culture conditions

The bacterial strains used in this research were *Escherichia coli* K12 strain MG1655, *Pseudomonas aeruginosa* (PA14), and methicillin-resistant *Staphylococcus aureus* (MRSA: ATCC 43300). For the growth of all bacterial strains, Luria–Bertani Broth (LB) broth and agar (composition: 1% tryptone (w/v), 0.5% yeast extract (w/v), and 1% NaCl (w/v), 2% agar/pH 7.2) were used. Cultures for all experiments were maintained at 37°C. The bacteriological chemicals utilized in this study were procured from Sisco Research Laboratories (SRL), Mumbai, India.

### Determination of the minimum inhibitory concentrations

The minimum inhibitory concentrations (MIC) of the antibiotic kendomycin were determined using the broth microdilution method as described previously by Wiegand et al. (2008). The microbroth dilution method was performed with transparent 96-well plate. Kendomycin was prepared at different working concentrations using a stock solution (0.5 mg/mL). A saline suspension of the test strain equivalent to 2.0 McFarland standard (containing 1 × 10^7^ to 1 × 10^8^ cfu mL^−1^) was prepared. A volume of 1–10 μL of the suspension was added to each well (to attain a final density of ~1 × 10^5^ cfu mL^−1^), and the cultures were incubated at 37°C for 12–16 h. The MIC was defined as the lowest concentration of a test antibiotic that completely inhibited visible bacterial growth.

### Microscopic analysis

Live cell imaging was performed as described previously (Govindarajan and Amster-Choder, 2017; Nagarajan et al., 2023). Actively growing cells were used for treatment and imaging. Overnight cultures were subcultured (1:100 dilution), and the cells were grown for 2 h at 37°C and then exposed to kendomycin (50 μg/mL) for 3–4 h before imaging. For the control, the cells were grown at 37°C for 5–6 h and imaged. The cells were harvested and stained with Synapto Red C2 (4 mg/mL) and DAPI (5 mg/mL) for 10 min. The cells were washed with 1× phosphate-buffered saline (PBS) and resuspended in 100–200 μL of PBS before imaging. In total, 10–20 μL of stained cells were spotted on 1% agarose pads (in 0.85% saline) with uncoated coverslips before visualization under a microscope. The cells were visualized and photographed using a Nikon Eclipse Ti2-E instrument equipped with a 100× CFI Plan Apochromat oil objective and a DSQi-2 Monochrome Camera (Nikon). Images were processed using NIS Elements AR software (Nikon).

### ADMAT analysis

The SMILES of each molecule were extracted from the PubChem database and converted into a 3D structure in PDB format using the online SMILES converter NCI/CADD. After that, the metabolites were produced by adding Gasteiger charges, non-polar hydrogen atoms, allocating aromatic carbons, and identifying the root using AutoDock Vina. After that, the ligands were stored in pdbqt format (Aragón-Muriel et al., 2021; Melinda et al., 2021). The SwissADME database was subsequently used to assess the drug-like characteristics of the active compounds. This is predicated on Christopher Lipinski’s discovery of Lipinski’s rule of 5. There are five parameters to it. The molecular weight should be within 500 Dalton, the value of logP should be <5, the number of bond donors should be H < 5, and the number of bond acceptors should be H > 10 (Naqvi et al., 2018).

## Results

### Isolation and characterization

**Figure 1:**
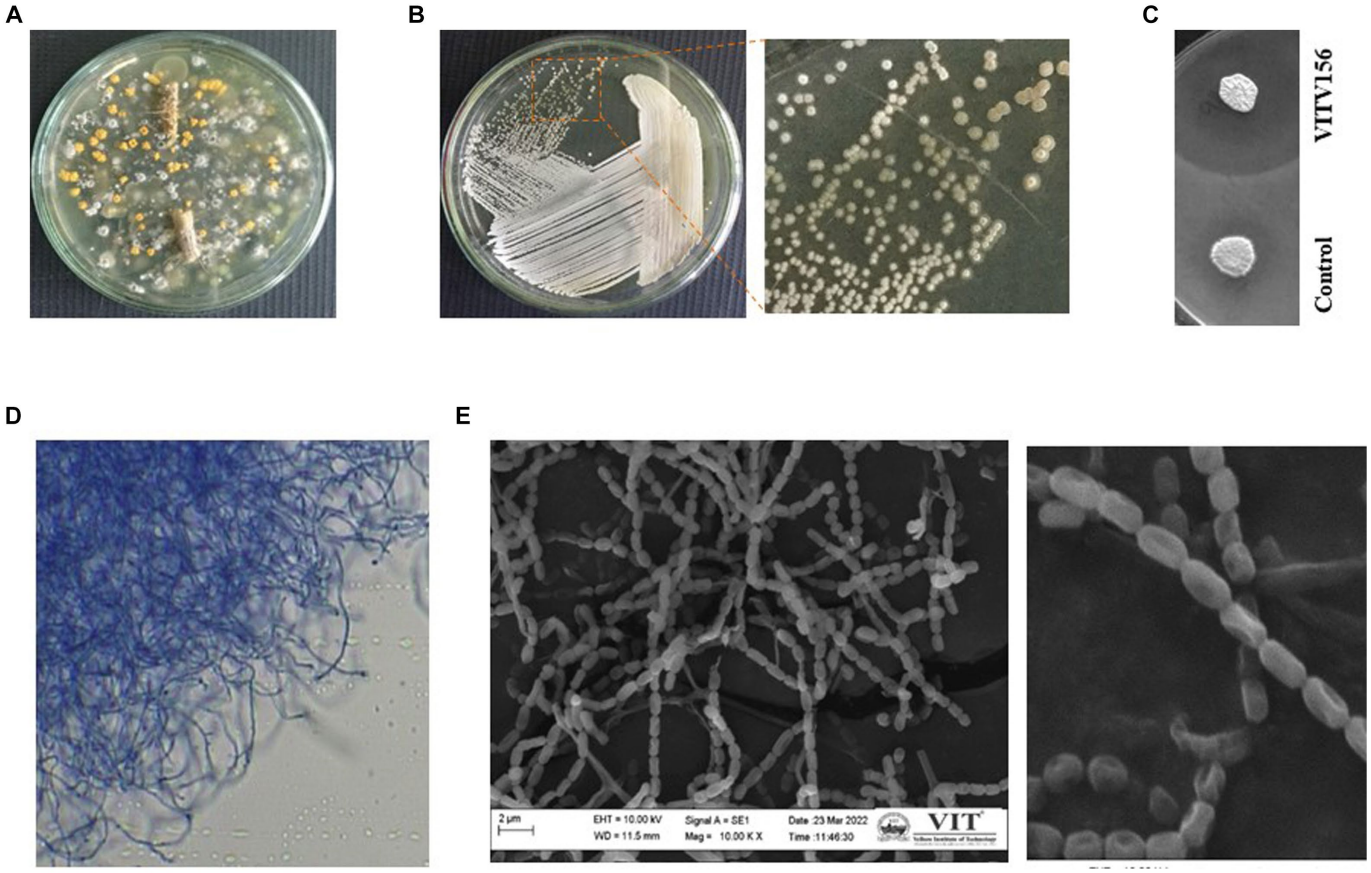
Isolation of *Streptomyces* sp. VITGV156. **(A)** Petri plate with a 7-day-old culture on ISP2 agar plate exhibiting diverse colony morphologies. **(B)** Colony morphology of *Streptomyces* sp. VITGV156. **(C)** VITGV156 and the control (another isolate with no antibacterial activity) were screened for antibacterial activity. Compared with the control, VITGV16 inhibited the growth of *Escherichia coli*. **(D)** Long, discontinuous hyphal growth was observed via phase contrast microscopy. **(E)** SEM image of the spore chain.

Our previous study (Veilumuthu and Christopher, 2022b) on analyzing the diversity of *Streptomyces* sp. endophytes in tomato plants revealed the hidden microbial treasures dwelling within these plants (Chanama et al., 2023). Endophytic *Streptomyces* sp. The production of diverse secondary metabolites was the focal point of this investigation. The practiced sample processing methodologies ensured the viability of endophytic species within the samples. A total of 240 endophytic actinomycetes were observed from the tomato plants taken for analysis, which is very evident from their distinct colony morphology. A visual representation of the endophytic *Streptomyces* sp. isolated from tomato plants on ISP2 agar plates is given in Figure 1A. Overall, Figure 1 shows the distinctive cultural attributes and colony morphology. Individual colonies were selected and purified by further streaking. A broad range of compounds produced by *Streptomyces* sp. were reported to exhibit antimicrobial activity. Therefore, a preliminary screening was performed by assessing the antimicrobial activity of the isolated strains by visualizing their activity against *Escherichia coli*. One of the isolates exhibited significant activity, exhibiting a large zone of clearance (Figures 1B,C). The strain was named *Streptomyces* sp. VITGV156 and characterized further.

Phenotypic characterization was performed to support the novelist VITGV156. Upon growth, they form aerial mycelia on the surface. The color of the substrate mycelium is pale yellow on ISP2. This complex mycelial composition was further characterized by clusters comprising both elongated and abbreviated fragments, as visually demonstrated in Figure 1D. A closer examination revealed an interconnected network of aerial hyphae. SEM images of the hyphae revealed delicate and weak chains of spores, with each spore featuring a prominent cavity on one side (Figure 1E). The spores measured approximately 1 μm in size.

### Genome features of *Streptomyces* sp. VITGV156

**Figure 2:**
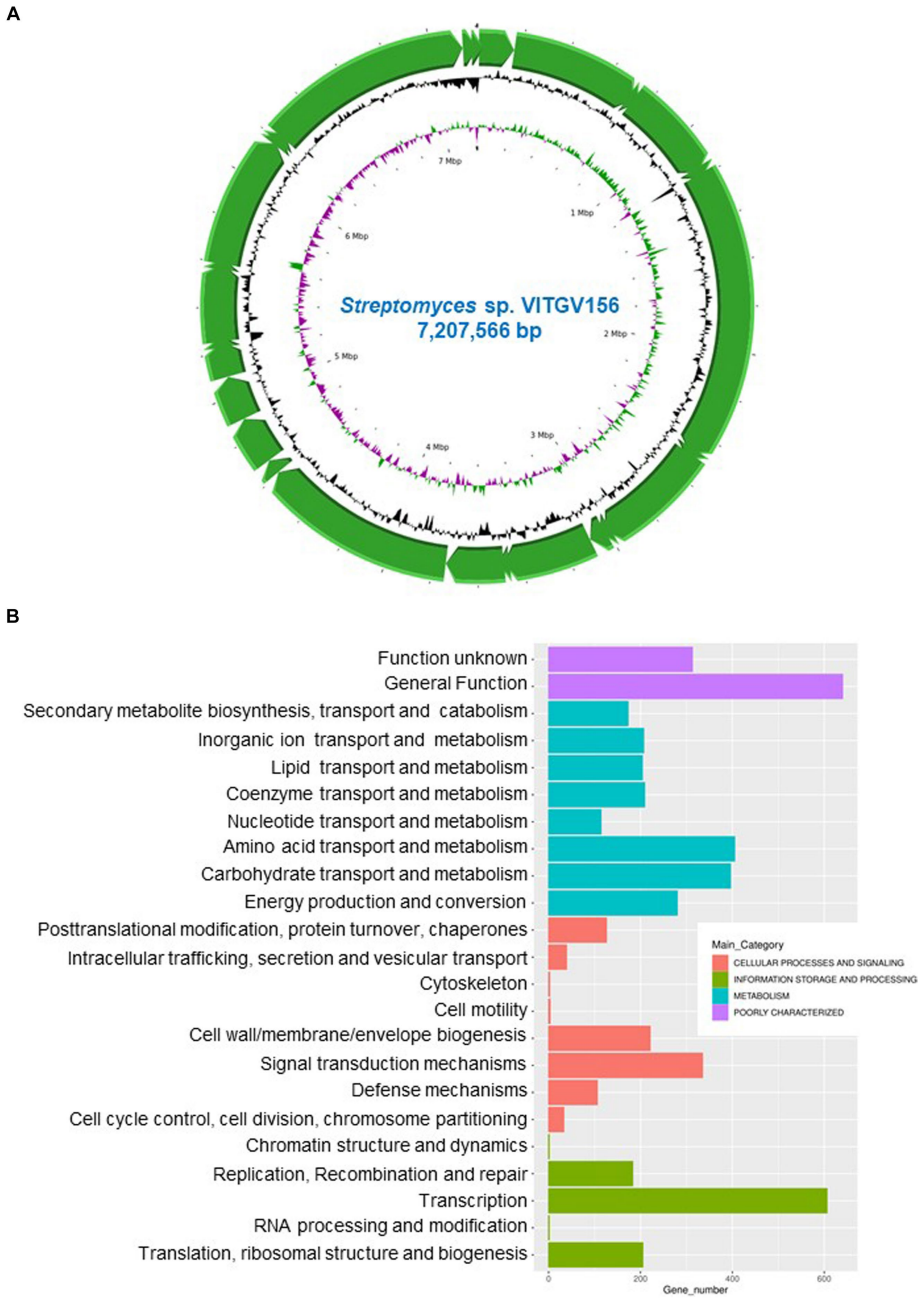
Genomic landscape of VITGV156. **(A)** Circular genomic map of *Streptomyces* sp. VITGV156. Assembled contigs are depicted as green arrows, accompanied by positive and negative GC skews indicated in the inner circle. **(B)** Cluster of Orthologous Gene (COG) Database annotation of *Streptomyces* sp. VITGV156. The graph shows the functional categorization of the *Streptomyces* sp. VITGV156-encoded ORFs into four main functional categories: information storage and processing, cellular processes and signaling, metabolism, and poorly uncharacterized. These categories are further subdivided into subcategories, as depicted by similarly colored bars along with their corresponding numbers.

Whole-genome sequencing was performed with genomic DNA extracted from *Streptomyces* sp. VITGV156 grown on ISP2 media for 15 days. High-quality genome sequencing was performed with the Illumina NextSeq 500 platform. The genome of *Streptomyces* sp. VITGV156 was successfully assembled into a complete chromosome measuring 7,207,566 bp, with a G + C content of 72.6% (Figure 2A). The genomic features of VITGV156 are detailed in Table 1. Within this genome, 6,583 predicted genes were identified, comprising 6,512 protein-coding genes and 20 rRNA-associated genes, including 67 tRNA genes (Supplementary material S1). Among the 6,512 encoded proteins, functions were assigned to 4,993 proteins (76.67%), while the functions of the remaining 1,519 proteins (23.32%) remained unknown and were annotated as hypothetical proteins. Notably, only 64.81% (4221) of the proteins from the VITGV156 genome were mapped to functional categories within the Clusters of Orthologous Groups (COGs) (Figure 2B), as detailed in Supplementary material S1. The genes were categorized into three functional categories: information storage and processing, cellular processes, and signaling metabolism.

**Table 1:** Genomic features of the strain *Streptomyces* sp. VITGV156.

S. No.	Feature	Value
1	Genome topology	Linear
2	Total genome size (bp)	8.18 Mb
3	Assembly size (bp)	7,207,566 bp
4	G + C content (%)	72.6
5	Coding DNA sequence	6,583
6	Protein coding genes	6,512
7	Unique genes (of unknown function)	1,519
8	Total no. of RNA operons	71
9	Total no. of tRNA operons	67
10	Total no. of rRNA operons	4
11	BioSample ID	20,499,087

### *Streptomyces* sp. VITGV156—comparative genome analyses

**Figure 3:**
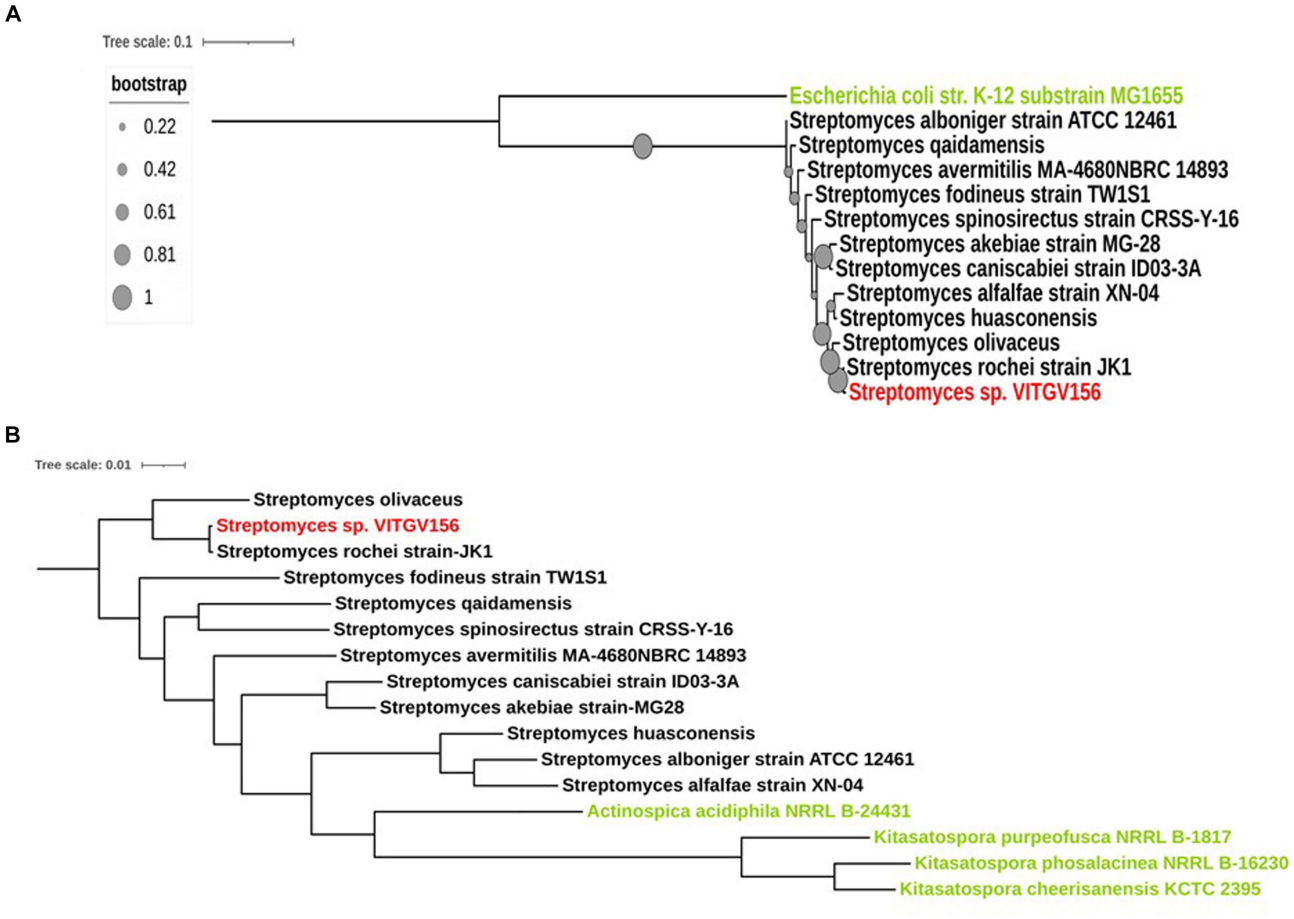
Phylogenetic analysis of *Streptomyces* sp. VITGV156. **(A)** 16S rRNA phylogenetic analysis of *Streptomyces* sp. VITGV156. The isolated strain of *Streptomyces*, denoted in red, shares a clade with the *Streptomyces rochei strain Jk1*. The outgroup is denoted in green. Bootstrap values are depicted as solid circles and annotated on the branches. **(B)** Phylogeny based on housekeeping proteins. Fourteen concatenated housekeeping proteins were used to generate a phylogenetic tree using AutoMLST and visualized with iTOL. *Actinospica acidiphila NRRL B-24431*, *Kitasatospora purpeofusca NRRL B-1817*, *Kitasatospora phosalacinea NRRL B-16230*, and *Kitasatospora cheerisanensis KCTC 2395* were used as outgroup species in this study (denoted in green).

The 16S rRNA sequence of *Streptomyces* sp. VITGV156 exhibited the highest similarity with the 16S rRNA sequence of *Streptomyces rochei* strain JK, followed by *Streptomyces huasconensis* strain D23 and *Streptomyces alfalfae* strain XN-04 (Figure 3A). Phylogenetic analysis based on 16S rRNA sequences also demonstrated that it forms a distinct clade alongside *Streptomyces fodineus* strain TW1S1, *Streptomyces spinosirectus* strain CRSS-Y-16, *Streptomyces caniscabiei* strain ID03-3A, *Streptomyces akebiae* strain MG28, *Streptomyces avermitilis* MA-4680 NBRC 14893, *Streptomyces alboniger* strain ATCC 12461, and *Streptomyces qaidamensis*, whereas *Escherichia coli* str K12 substrain MG1655 was taken as an outgroup to denote one of the most distant relatives of the isolated species of *Streptomyces*. To further validate the phylogeny, we performed multilocus sequence analysis (MLSA) (Macheras et al., 2011) using housekeeping genes. Therefore, we conducted a housekeeping gene analysis to validate the taxonomic relationships among *Streptomyces* species. Specifically, we analyzed the gene sequences of *atpD*, *gyrB*, *recA*, *rpoB*, and *trpB*, which are commonly used in multilocus sequence analysis (MLSA) (Komaki, 2022) for *Streptomyces* (Figure 3B). We further included sequences from nine more conserved genes for MLST analysis of this strain (*tigrfam_recA*, *rpsJ_bact*, *uS11_bact*, *ftsZ*, *rpsS_bact*, *rplN_bact*, *rpoC_TIGR*, *rpsE_bact*, and *rpsL_bact*) (Supplementary Table S1). Our phylogenetic analysis, which included 14 housekeeping genes from *Streptomyces* and five outgroups (Figure 3B), yielded a tree topology similar to that obtained using 16S rRNA. This analysis supports the assertion that strain VITGV156 is closely related to *S. rochei* JK.

**Figure 4:**
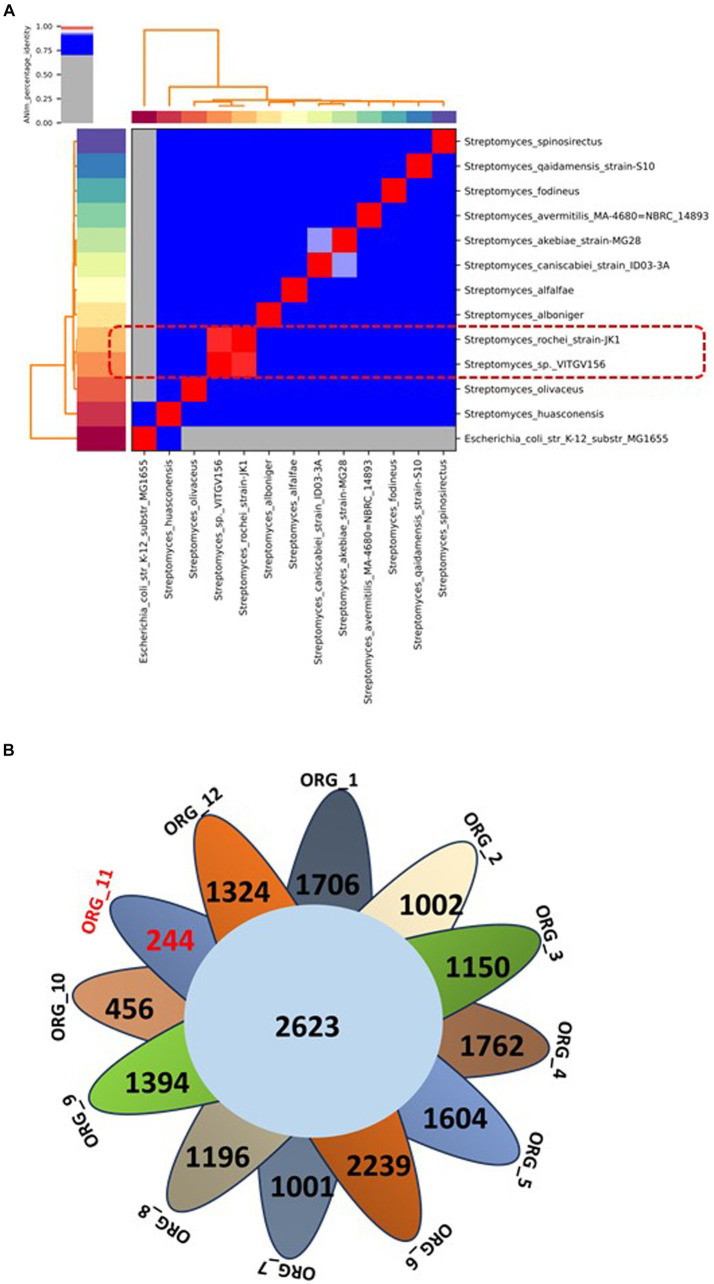
**(A)** Average nucleotide identity (ANI) of *Streptomyces* compared with 11 other complete genomes of *Streptomyces* species. The highest score is highlighted in red within the matrix. The ANI (98.94%) of the isolated strain and one of its closest relatives are indicated in the box in Red dotted line. **(B)** A Venn diagram showing the distribution of core and unique genes among the 12 genomes of the *Streptomyces* genus. The number of unique genes in *Streptomyces* sp. VITGV156 is highlighted in red (Org_11).

To support this analysis, we computed the *in silico* DDH values of *Streptomyces* sp. VITGV156 compared to those of these *Streptomyces* strains using the Genome-to-Genome Distance Calculator server. We found that the highest DDH value of 95.63 was exhibited by the *Streptomyces rochei* strain JK1 (Supplementary material S1). Furthermore, we calculated the average nucleotide identity (ANI) for these genomes, revealing that *Streptomyces* sp. VITGV156 shares 98.94% similarity with the *Streptomyces rochei* strain JK1 (Figure 4A). These findings are consistent with the 16S rRNA phylogenetic analysis, indicating that the isolated strain of *Streptomyces* is the closest relative to the species *Streptomyces rochei*.

The panproteome encompasses all proteins shared among more than two species and comprises the core proteome, dispensable proteome, and unique proteome. The collective proteome of 12 *Streptomyces* genomes comprises 91,301 proteins distributed into 12,293 orthologous protein clusters (see Supplementary material S2). Among these clusters, 2,623 were conserved across all genomes; constituting the core proteome for these *Streptomyces* strains (Figure 4B, Table 2). Additionally, 9,671 and 14,351 clusters were categorized as accessory and unique genes, respectively (see Supplementary material S2). This analysis identified 270 unique genes in the *Streptomyces* sp. VITGV156 strain, 207 of which are hypothetical and are documented in Supplementary material S2.

**Table 2:** Pangenome of *Streptomyces* genomes highlighting core and unique genes in genomes.

S. No	Name	Species	Core gene clusters	Unique gene clusters
1	Org_1	*Streptomyces akebiae* MG28	2,623	1,585
2	Org_2	*Streptomyces alboniger*	2,623	868
3	Org_3	*Streptomyces alfalfa*	2,623	1,046
4	Org_4	*Streptomyces avermitilis* MA-4680 NBRC14893	2,623	1,676
5	Org_5	*Streptomyces caniscabiei* ID03-3A	2,623	1,489
6	Org_6	*Streptomyces fodineus*	2,623	2050
7	Org_7	*Streptomyces huasconensis*	2,623	834
8	Org_8	*Streptomyces olivaceus*	2,623	1,071
9	Org_9	*Streptomyces qaidamensis* S10	2,623	1,275
10	Org_10	*Streptomyces rochei* JK1	2,623	424
11	Org_11	*Streptomyces* sp. VITGV156	2,623	270
12	Org_12	*Streptomyces spinosirectus*	2,623	1,200

Our comparative analysis of the *Streptomyces* sp. VITGV156 (Org_11) strain revealed its novelty, identifying it as the closest relative to *S. rochei* (Org_10). Within its genome, we identified 270 unique genes, 76.66% of which were entirely novel. Annotation data indicate that these genes are hypothetical, lacking known domains, and potentially encoding novel functions that are not observed in any other organism. This isolation distinguished this newly discovered species from other *Streptomyces* species. We report its genomic characterization through comparative genomic analyses. The VITGV156 strain was submitted to the National Center for Microbial Resources (NCMR) under accession number MCC4965.

### The genome of VITGV156 encodes a wide range of polyketide antibiotics

To identify the gene clusters responsible for the production of putative secondary metabolites, the genome sequence of VITGV156 was analyzed using the antiSMASH software. The genome of VITGV156 encodes 29 putative biosynthetic gene clusters (BGCs). Eight compounds showed relatively low similarity (<20%) to known natural products, suggesting that they are unidentified compounds. Of the 29 BGCs found to be responsible for the production of secondary metabolites, 65% (16 BGCs) corresponded to the biosynthesis of peptide compounds, which included non-ribosomal peptide synthetases (NRPSs) and ribosomally synthesized and posttranslationally modified peptides (RiPPs). NRPSs are large, multidomain enzymes found in bacteria and fungi that are responsible for the biosynthesis of complex peptides. Unlike ribosomal protein synthesis, NRPSs catalyze peptide biosynthesis in a template-independent manner. The peptides produced by NRPs exhibit a wide range of biological activities and thus have antimicrobial and anticancer properties (Sieber and Marahiel, 2005; Strieker et al., 2010; Martínez-Núñez and López, 2016). The genome of VITGV156 encodes a calcium-dependent antibiotic gene (CDA1/CDA2a). CDA1 and CDA2a are enzymes involved in the biosynthesis of CDAs. These enzymes are responsible for catalyzing specific steps in the synthesis of the CDA molecule. The exact functions of CDA1 and CDA2a may vary depending on the specific reactions they catalyze within the biosynthetic pathway. The antimicrobial activity of CDAs is well known in Gram-positive bacteria (Micklefield, 2009; Wood and Martin, 2019). RiPPs are peptides synthesized by ribosomes in a template-dependent manner and are modified posttranscriptionally by modifying enzymes. RiPPs are known for their remarkable biological activities (antimicrobial, anticancer, and immunomodulatory activities) (Papini, 2009; Ongpipattanakul et al., 2022). In addition, 17% of BGCs (4) corresponded to the synthesis of terpenes, and 10% of BGCs (3) corresponded to the synthesis of siderophores. The indole compound 5-dimethylallylindole-3-acetonitrile was also produced by the strain (Table 3).

**Table 3:** Secondary metabolite BGC present in VITGV156, identified using antiSMASH 7.0.1.

S. No.	Scaffolds	Type	Compound	Similarity (%)
1	Region 2.1	Indole	5-Dimethylallylindole-3-acetonitrile	100
2	Region 3.2	T3PKS	Flaviolin/1,3,6,8-1,3 tetrahydroxy naphthalene	100
3	Region 7.1	Ectoine	Ectoine	100
4	Region 7.3	NI siderophore	Desferrioxamine B	100
5	Region 12.1	Terpene	Albaflavenone	100
6	Region 17.2	Terpene	Geosmin	100
7	Region 18.2	Terpene	Hopene	100
8	Region 21.4	NRP metallophore, NRPS	Coelichelin	100
9	Region 21.6	Class III lanthipeptide	Sap B, RiPP: Lanthipeptide	100
10	Region 3.1	NRPS-like	Streptothricin	95
11	Region 21.5	NRP metallophore, NRPS, class I lanthipeptide	Coelibactin	90
12	Region 18.4	T1PKS	Vicenistatin	70
13	Region 18.1	NRPS	CDA1/CDA2a	72
14	Region 13.1	T2PKS, PKS-like	Fluostatins M-Q	67
15	Region 12.2	T2PKS	Spore pigment	66
16	Region 2.2	Terpene	Isorenieratene	63
17	Region 11.1	Class III lanthipeptide	Catenulipeptin	60
18	Region 21.3	RiPP-like	Informatipeptin	42
19	Region 20.1	T1PKS	Stambomycin	32
20	Region 18.3	Hydrogen cyanide	Aborycin	28
21	Region 21.1	T1PKS	Streptovaricin	29
22	Region 16.1	NI siderophore	Kinamycin	16
23	Region 19.1	T1PKS	Kendomycin B	15
24	Region 17.3	NI siderophore	Paulomycin	11
25	Region 10.2	PKS-like, furan, class V lanthipeptide	Methylenomycin A	9
26	Region 15.1	Butyrolactone	Prejadomycin, Rabelomycin, Gaudimycin C	6
27	Region 10.1	Polyketide: Type II polyketide	Granaticin	5
28	Region 21.2	Terpene	Versipelostatin	5
29	Region 7.2	Melanin	Istamycin	4

Moreover, the genome of VITGV156 harbors genes responsible for the biosynthesis of at least 11 polyketide antibiotics (italicized in Supplementary Table S2). Polyketide compounds are a diverse class of naturally derived compounds that are prominent for their bioactivity, including antibiotics, immunosuppressants, and anticancer chemotherapeutics (Gomes et al., 2013; Tacar et al., 2013; Risdian et al., 2019). Interestingly, the BGCs encoding polyketide antibiotics were less similar to the existing BGCs, indicating that they were hitherto unidentified.

To determine the efficiency of the predicted polyketide compounds, we analyzed their drug-likeness properties, as per the AntiSMASH report. The detailed list is given in Supplementary Table S3. The molecular weights of the compounds were found to be in the range of 182–1,375 g/mol. Of the 10 compounds tested, three did not obey Lipinski’s rule, as there was more than 1 violation, and seven of these compounds obeyed Lipinski’s rule, demonstrating their drug-like properties. In this study, we further investigated the potential antimicrobial activity of the polyketide antibiotic kendomycin (Elnakady et al., 2016).

### Kendomycin biosynthetic clusters of VITGV156

Kendomycin is a polyketide antibiotic known for its anticancer and antibacterial properties (Elnakady et al., 2007). The BGCs identified in the genome of VITGV156 were analyzed for kendomycin biosynthesis clusters. The VITGV156 genome encodes approximately five type I PKS gene clusters, which form the polyketide backbone of kendomycin and are similar to those of *S. verrucosispora* and *S. violaceoruber*. The genes encoding the T1PKS-kendomycin biosynthesis-related gene cluster span approximately 30.02 kb and encode 20 open reading frames (ORFs), akin to the BGCs found in *S. verrucosispora* and *S. violaceoruber*. However, we could not find any other enzymes (such as modifying, tailoring, and cycling enzymes in the kendomycin biosynthesis pathway). Notably, the presence of PKS genes and associated proteins in *Streptomyces* sp. VITGV156 indicates their involvement in various biosynthetic pathways, such as the 3,5-DHPG pathway, b-ketoadipate pathway, and tricarboxylic acid cycle. The kendomycin type I PKS assembly line comprises four enzymes encoded by a putative operon, with eight elongation modules incorporating extender units. Initiation involves activation of a likely benzoic acid derivative starter unit by a CoA ligase domain, while elongation is facilitated by specific enzymes assigned to different modules based on domain analysis. Further investigations are warranted to fully elucidate the complexities of kendomycin biosynthesis.

### Kendomycin treatment affects membrane and nucleoid organization

**Figure 5:**
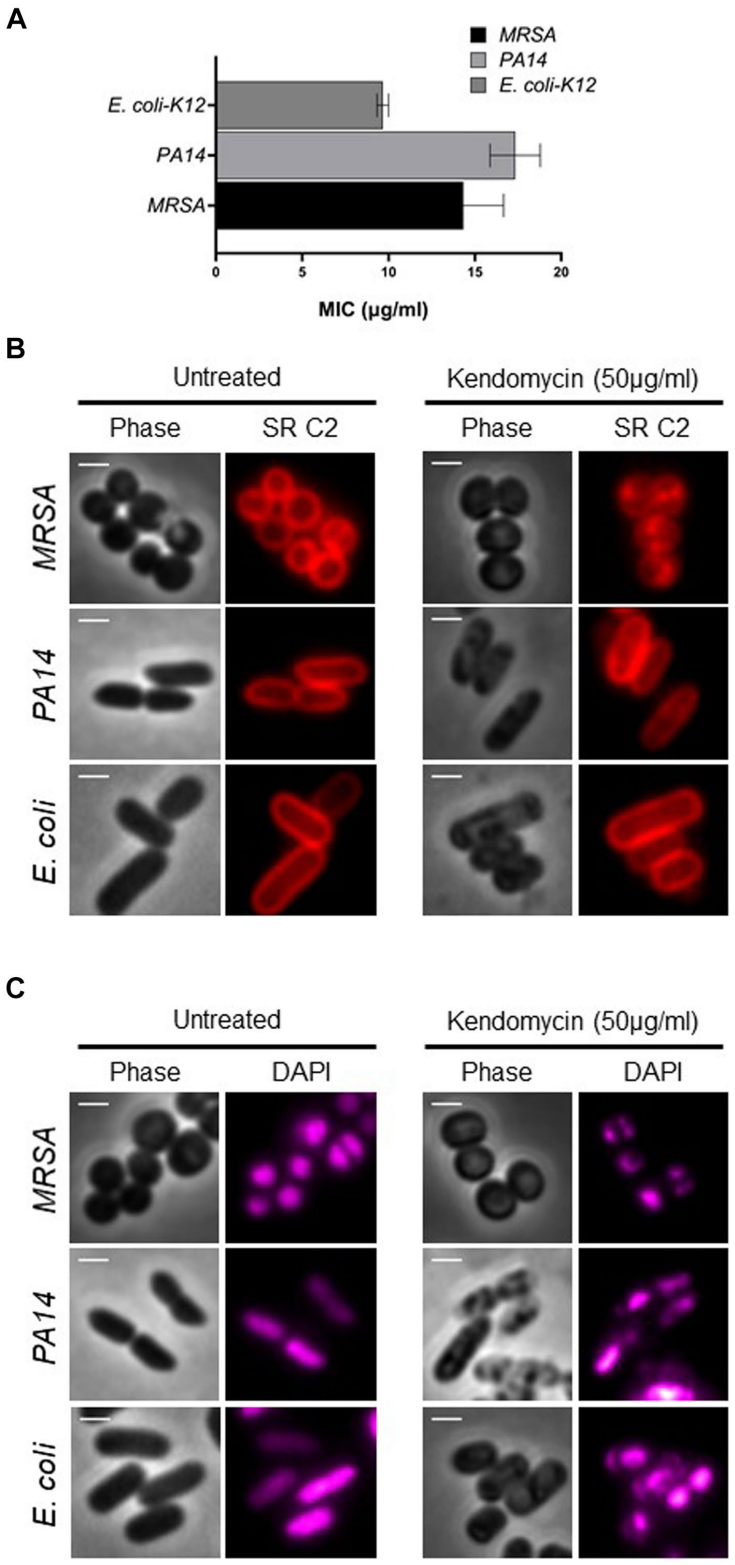
Antimicrobial activity of Kendomycin. **(A)** Minimum inhibitory concentration of kendomycin for the indicated bacteria. **(B)** Images of the indicated bacterial cells exposed to different concentrations of kendomycin (50 μg/mL) and membrane stained. The bacterial membrane was observed by fluorescence microscopy, and the cells were observed via phase contrast microscopy. Phase contrast (gray) and Synapto Red C2 (red) are shown. The scale bar corresponds to 2 μm. **(C)** Images of the indicated bacterial cells after exposure to different concentrations of kendomycin (50 μg/mL) and nucleoid staining. Bacterial nucleoids were observed by fluorescence microscopy, and the cells were observed via phase contrast microscopy. For better visualization, DAPI is shown in magenta. Phase contrast (gray) and DAPI (magenta) are shown. The scale bar corresponds to 2 μm.

We then aimed to elucidate the toxicity induced by kendomycin, a polyketide antibiotic. The antimicrobial property of kendomycin has drawn attention due to its promising activity against drug-resistant bacteria. To ascertain the toxicity of kendomycin, we assessed the minimum inhibitory concentration for Gram-positive [methicillin-resistant *S. aureus* (MRSA)] and Gram-negative (*P. aeruginosa* and *E. coli*) bacteria through the broth microdilution method (Wiegand et al., 2008). The inhibitory concentrations of kendomycin for MRSA, *P. aeruginosa*, and *E. coli* ranged from 10 to 15 μg/mL (Figure 5A). We further extended the study by evaluating the bacterial morphology after brief exposure (4–6 h) to high concentrations of kendomycin (50 μg/mL). Microscopic visualization of kendomycin-treated bacteria that exhibited severe damage to the membrane. The bacterial membrane exhibited abnormalities, which were visualized both by phase contrast and with a fluorescent red filter (stained with SynaptoRed C2). Phase contrast images of Gram-negative bacteria revealed severe irregularities on the cell surface, whereas SynaptoRed C2 staining revealed distinct puncta patches in the cell membrane in MRSA (Figure 5B). These results indicated possible damage to the bacterial membrane upon kendomycin exposure. Previous studies on kidney toxicity reported global changes in the proteome and transcriptome (Duangmal et al., 2016). Since bacterial nucleoids are associated with the expression patterns of different genes (Kar et al., 2005; Jin and Cabrera, 2006; Dillon and Dorman, 2010), we assessed the nucleoid morphology of the bacterial cells before and after treatment with 4′,6-diamidino-2-phenylindole (DAPI), a DNA-staining dye. The results indicated a drastic change in the nucleoid morphology in all the bacterial strains tested. We observed a reduction in the size of the nucleoid along with morphological changes in the nucleoid after treatment (Figure 5C). Taken together, our results indicated that kendomycin treatment potentially causes damage to cells by affecting various targets.

### LC-HRMS analysis of isolated compounds

**Figure 6:**
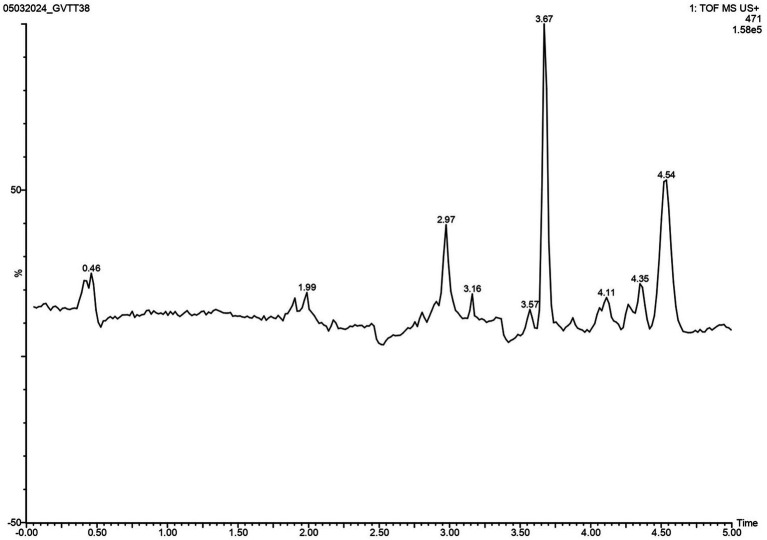
LC–MS/MS chromatogram of the secondary metabolite of *Streptomyces* sp. VITGV156. The peak corresponds to the bioactive molecule kendomycin (peak at 3.637, *m/z* = 470.3007).

**Figure 7:**
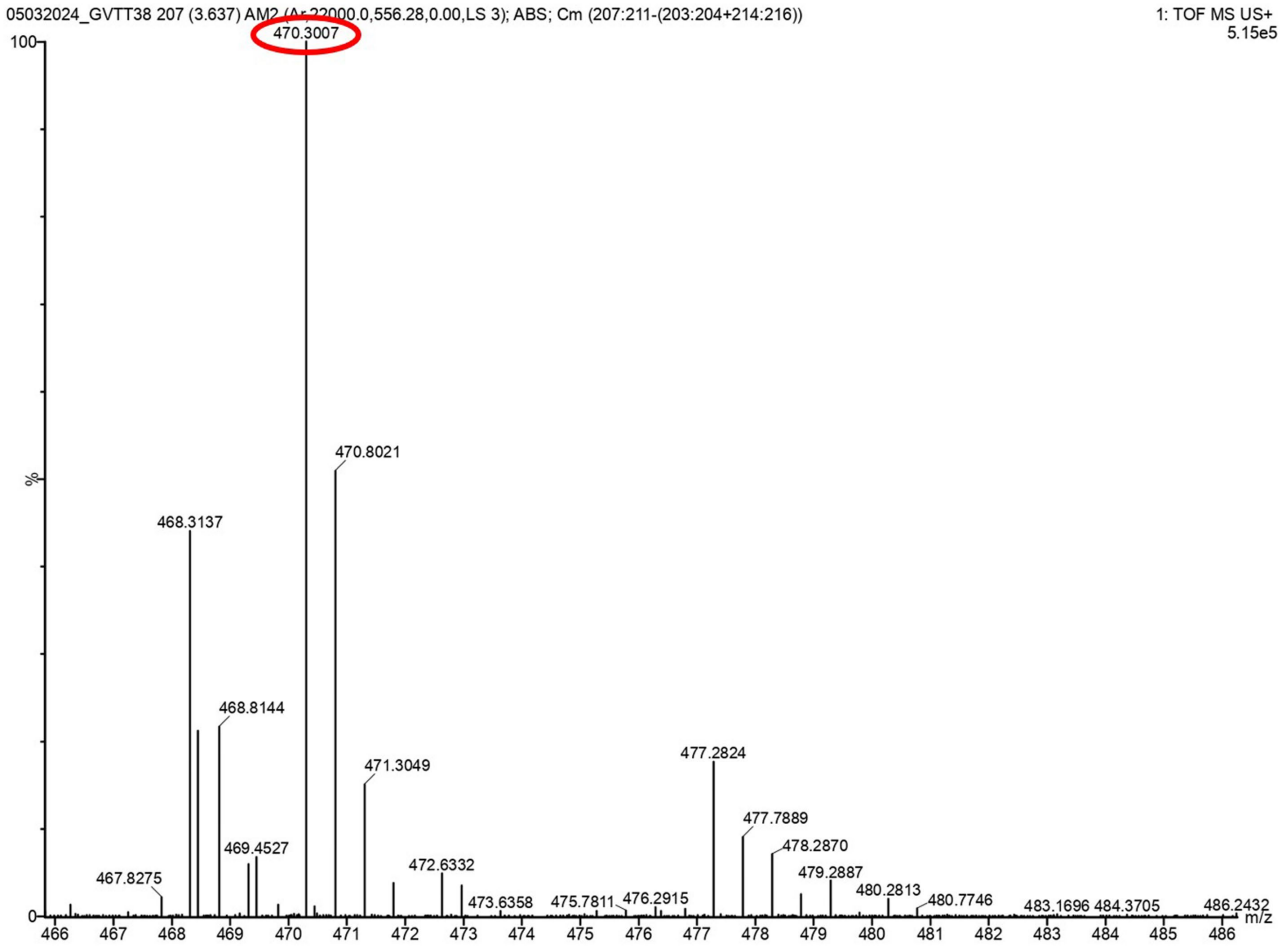
LC-HRMS-ESI spectrum of the compound kendomycin. The retention time was 3.637 min, and the mass of the spectrum is 470.3007.

The task of isolating metabolites presents significant challenges. To identify these metabolites, LC-HRMS analysis was done for VITGV156 crude extracts. The LC-HRMS data were scrutinized utilizing m/z, retention time, and molecular formula, with additional databases utilized to search for and assign formulas and compound structures. Following evaluation and interpretation, the compound identified at a retention time (RT) value of 3.63 was determined to be kendomycin (Figure 6). The molecular formula and molecular mass of these identified compounds are C_29_H_42_O_5_ and 470.3007, respectively (Figure 7).

## Discussion

*Streptomyces* sp. are ubiquitously present in the soil and are associated with different flora. Endophytic *Streptomyces*, which are symbiotic with the host, offer many benefits to the host, including the production of plant growth-promoting factors and the inhibition of the growth of pathogenic bacteria (as biocontrol agents) (Vurukonda et al., 2018). Additionally, endophytic associations confer endophytic bacteria with the ability to produce host metabolites by establishing deeper connections (Li et al., 2022). Therefore, the isolation and characterization of endophytic *Streptomyces* are more advantageous than the use of free-living *Streptomyces*.

In terms of global dietary preferences, tomatoes are a prominent and widely consumed plant-based food. Renowned for their rich repository of clinically significant bioactive compounds, these fruits hold immense nutritional value. An intriguing facet of tomato plants lies in their symbiotic association with an array of endophytic microbes (Chopra and Roberts, 2001; Martínez-Núñez and López, 2016; Santoyo et al., 2016; Li et al., 2022). This study aimed to isolate *Streptomyces* sp. endophytic from tomato plants, which have high potential to produce secondary metabolites of nutritional and commercial value. Our attempt to isolate *Streptomyces* endophytes from tomato plants resulted in the identification of a novel strain, *Streptomyces* sp. VITGV156. The strain exhibited significant antibacterial activity and exhibited potential for secondary metabolites. The observed colony and spore of VITGV156 resemble the characteristics of *Streptomyces* sp. and are on par with the findings of various other studies (Aouar et al., 2012; Bhandari et al., 2022).

Whole-genome sequencing helps in the comprehensive analysis of the VITGV156 genome, the identification and classification of the isolate, and bioprospecting for potential secondary metabolite gene clusters. The isolated *Streptomyces* sp. VITGV156 is phylogenetically most closely related to *S. rochei* and has a remarkable resemblance in terms of cellular morphology (Pazhanimurugan et al., 2016). Consistent with these results, another endophytic species, *Streptomyces* sp. PT2, which is isolated from tomato plants, exhibits high similarity with *S. rochei* (Singh and Dubey, 2018). Several species of *S. rochei* have been isolated from various niches, including the rhizosphere and terrestrial ecosystem, and have wide applications (Kanini et al., 2013; Hamdan et al., 2021; Zhou et al., 2024). Bioprospecting for antimicrobial secondary metabolites revealed a staggering count of 29 biosynthesis-related gene clusters, of which 17 gene clusters exhibited noteworthy resemblances to other existing BGCs (similarity above 50%). The genome encompasses several BGCs for the production of polyketide antimicrobial compounds, such as vicenistatin, the fluostatin M-Q, stambomycin, streptovaricin, kanamycin, kendomycin B, methylenomycin A, prejadomycin, rabelomycin, gaudimycin C, granaticin, versipelostatin, and istamycin (Table 2). This finding indicates that VITGV156 is fertile ground for the emergence of novel derivatives and bioactive compounds. These compounds have been previously identified in various vicenistatin strains of *Streptomyces halstedii* (Chopra and Roberts, 2001); the fluostatin M-Q in *Streptomyces* sp. PKU-MA00045 (Jin et al., 2018); the stambomycin in *Streptomyces ambofaciens* (Laureti et al., 2011); the kendomycin B in *Streptomyces* sp. Cl 58–27 (Paulus et al., 2021); the methylenomycin A in *Streptomyces violaceusruber* SANK 95570 (Aguilar and Hopwood, 1982); the buyrolactone compounds in *Streptomyces chattanoogensis* (Du et al., 2011); the versipelostatin in *Streptomyces versipellis* 4,083-SVS6 (Ueda et al., 2008); and the istamycin in *Streptomyces tenjimariensis* ATCC31603 (Hoang et al., 2016). Notably, many BGCs exhibit minimal similarity, below 50% (4), and a significant number of BGCs exhibit similarities, below 20% (8), further indicating that *Streptomyces* sp. VITGV156 is a potent producer of diverse bioactive secondary metabolites. Remarkably, most of the BGCs encoding polyketide antibiotics are <20%, indicating that they could be derivates of this class of antibiotics, which is worth investigating. The genome of VITGV156 contains genes responsible for the production of a different class of antimicrobial compounds, which includes non-ribosomal peptide synthetases (NRPSs) and ribosomally synthesized and posttranslationally modified peptides (RiPPs). NRPSs are complex multimodular enzymes that produce non-ribosomal peptides (NRPs), which have diverse biological applications (Strieker et al., 2010; Martínez-Núñez and López, 2016). Many non-ribosomal peptides have antimicrobial activity, and many serve as last-resort antimicrobial agents (Liu et al., 2019). Notably, at least five different NRPSs (Table 2) encode VITGV156, one of which is a well-known antibacterial agent, Streptothricin (Morgan et al., 2023). The products of other NRPSs remain elusive. RiPPs are ribosomal-synthesized posttranslationally modified antimicrobial peptides of commercial value. Owing to their broad range of antibacterial activity against Gram-positive and Gram-negative bacteria, they are commonly used in the food and pharmaceutical industries (Van Kraaij et al., 1999; Arnison et al., 2013). The genome of VITGV156 encodes two RiPPs, two of which exhibit low sequence similarity with known compounds (Table 2). These results are consistent with the results of previous studies on *Streptomyces* sp. Babs14, which exhibited 29 biosynthetic secondary metabolite gene clusters, of which eight gene clusters displayed 100% congruence with known clusters (Zerouki et al., 2021). An earlier investigation (Cruz-Morales et al., 2017) identified secondary metabolites such as siderophores in *Streptomyces* sp. CC77. This finding adds to the broader understanding of secondary metabolite diversity within the *Streptomyces* genus. Analogous findings have been reported across several *Streptomyces* species; for instance, *Streptomyces* sp. ICC1 boasts a compilation of 37 BGCs (Gosse et al., 2019). Additionally, the genome of VITGV156 also revealed the presence of genes responsible for the biosynthesis of butyrolactone, siderophores, indoles, ectoine, and terpenes.

Notably, *Streptomyces* sp. VITGV156 has BGCs for many polyketide antibiotics. They are characterized by their complex structures, which contain multiple cyclic rings, conjugated double bonds, and various functional groups (John, 1991). Interestingly, these compounds exhibit broad-spectrum antimicrobial activity and have been shown to be effective even against drug-resistant pathogens (Kizhakkekalam et al., 2020; Chakraborty et al., 2021). They are synthesized by multimodal enzyme complexes called polyketide synthases (PKSs). Simple building blocks such as acetyl-CoA and malonyl-CoA are iteratively condensed to form a polyketide chain, which is subsequently processed by a group of tailoring enzymes. These enzymes include ketoreductases (KRs), dehydratases (DHs), enoyl reductases (ERs), and methyltransferases (MTs), which modify the polyketide backbone. This process results in the production of a vast array of structurally diverse molecules. Finally, the modified polyketide chain undergoes a cyclization reaction to form the characteristic pentacyclic core structure (Chen and Du, 2016; Ray and Moore, 2016; Xu and Arimoto, 2016; Wang et al., 2020). Among these antibiotics, the antibiotic class kendomycin has attracted attention for the following reasons. Kendomycin has broad-spectrum antimicrobial activity and has been shown to affect various cellular processes upon exposure (Elnakady et al., 2007, 2016; Tranter et al., 2020), making it a suitable candidate for the treatment of multidrug-resistant pathogens. However, kendomycin is produced by a relatively small number of *Streptomyces* sp. strains, making it rare (Xu and Arimoto, 2016; Chen et al., 2021; Paulus et al., 2021). Additionally, the biosynthetic pathway of kendomycin biosynthesis has not been fully elucidated. The genome of VITGV156 harbors five Type I PKSs corresponding to the biosynthesis of kendomycin. However, the details of their structure and enzymatic capability remain less explored. The identification of kendomycin B BGC through whole-genome sequencing and subsequent bioinformatics analyses has provided a foundational understanding of the genetic architecture driving kendomycin synthesis in *Streptomyces* sp. VITGV156. Notably, the presence of a type I polyketide synthase (PKS) gene cluster, sharing significant similarity with known kendomycin BGCs in *Verrucosispora* sp. and *Streptomyces violaceoruber*, underscores the conservation of kendomycin biosynthetic pathways across different strains. In addition, other enzymes of the kendomcyin biosynthesis pathway, such as modifying and cyclization enzymes need to be identified, which forms the future scope of this study.

To correlate the relevance of this study to plant pathogen control, many citations are there: *Streptomyces griseus* H7602 and *Streptomyces griseorubens* E44G are associated with the control of *Phytophthora capsici* and *Fusarium oxysporum f.* sp. *lycopersici*, both devastating pathogens of tomato plants (Khan et al., 2023). The ecological significance of these findings lies in the potential of these *Streptomyces* strains to offer sustainable solutions for disease management in tomato cultivation. By harnessing the beneficial effects of these endophytic bacteria, farmers can reduce their reliance on chemical pesticides and promote environmentally friendly agricultural practices.

Our attempt to understand the molecular basis of the antimicrobial activity of kendomcyin B revealed that kendomycin caused multifaceted damage in both Gram-positive and Gram-negative bacteria. Our studies revealed a striking pattern of damage induced by kendomycin B in bacteria, with membrane damage accompanied by nucleoid structure remodeling. Bacterial cellular processes are intricately associated with the cell membrane owing to the lack of cellular organelles (Govindarajan and Amster-Choder, 2016). Therefore, the bacterial membrane is highly dynamic, heterogeneous in composition, and allows lateral diffusion of membrane proteins to facilitate cellular processes (Vereb et al., 2003). Therefore, it is plausible that kendomycin-induced toxicity is due to the inhibition of various cellular processes associated with the bacterial membrane and nucleoid. Kendomycin exposure disrupts the cell division process by interfering with cell septum formation in a manner similar to penicillin (Elnakady et al., 2016). Overall, our exploration of endophytic *Streptomyces* resulted in the isolation of *Streptomyces* sp. VITGV156, which has remarkable bioprospecting potential for antimicrobial compounds.

## Conclusion

In this study, we endeavored to isolate endophytes. *Streptomyces* spp. are capable of producing novel antibiotics from tomato plants. Our studies resulted in the isolation of *Streptomyces* sp. VITGV156 from healthy tomato plants. The whole-genome sequencing of VITGV156 revealed its genomic landscape and its striking similarity to that of *S. rochei*. Additional analysis revealed the presence of several BGCs capable of producing polyketide antibiotics. Our analysis of kendomycin biosynthetic clusters revealed five Type I polyketide synthases: *S. verrucosispora* and *S. violaceoruber*. Furthermore, we extended our analysis and showed that kendomycin B causes damage to the membrane, changes the nucleoid morphology, and possibly can bind to membrane proteins during bacterial killing.

## Data availability statement

The whole genome of *Streptomyces* sp. VITGV156 was submitted to the NCBI SRA repository under BioProject accession number PRJNA750872 (https://www.ncbi.nlm.nih.gov/bioproject/PRJNA750872), BioSample accession number SAMN20499087 (https://www.ncbi.nlm.nih.gov/biosample/20499087), SRA accession number SRS9645416 and Accession ID: 20499087 & NCMR (Culture Deposit Accession no)—MCC4965.

## Author contributions

PV: Writing – original draft, Investigation, Methodology, Writing – review & editing. TN: Writing – review & editing, Data curation, Formal analysis, Resources. SM: Data curation, Formal analysis, Writing – review & editing, Validation. SS: Validation, Writing – review & editing, Conceptualization, Investigation, Methodology. LM: Investigation, Methodology, Writing – review & editing, Resources. TT: Writing – review & editing, Conceptualization, Supervision, Validation. JC: Conceptualization, Supervision, Writing – review & editing, Resources, Writing – original draft.

## Footnotes

^1^ https://ftp.ncbi.nih.gov/pub/COG/COG2020/data/
