# Supplementary material for: Genomic insights into an endophytic Streptomyces sp. VITGV156 for antimicrobial compounds
Source: Front Microbiol. 2024 Jun 3;15:1407289. doi: 10.3389/fmicb.2024.1407289 (PMC11180775; doi:10.3389/fmicb.2024.1407289)
Supplement: Supplementary file 1 [file Table_1.DOCX]

**Exploring the Genome of a Novel Endophyte, *Streptomyces* sps VITGV156 - Unraveling Pathways for Antimicrobial Compound Synthesis**

**Veilumuthu Pattapulavar^1^, Nagarajan T^2^, Sharayu Magar^2^, Sasikumar Sundaresan^3^, Lenus Joy Moses^1^, Thomas Theodore^4^, John Godwin Christopher^1^***

^1^Department of Biomedical Sciences, School of BioSciences and Technology, Vellore Institute of Technology, Vellore – 632014, India; ^2^Department of Biological Sciences, SRM University–AP, Andhra Pradesh – 522502, India; ^3^Department of Biochemistry, School of Biological Sciences, Madurai Kamaraj University, Madurai – 652021, India; ^4^School of Chemical Engineering, Vellore Institute of Technology, Vellore – 632014, India.

**Supplementary Information**

**Table S1**. List of housekeeping genes taken for MLSA analysis

| **Gene ID** | **Gene name** | **Functional category** | **Function** |
| --- | --- | --- | --- |
| TIGR01039 | *atpD* | Energy metabolism | ATP synthase F1, beta subunit |
| TIGR01059 | *gyrB* | DNA metabolism | DNA gyrase, B subunit |
| TIGR02386 | *rpoC_TIGR* | Transcription | DNA-directed RNA polymerase, beta' subunit |
| TIGR02012 | *tigrfam_recA* | DNA metabolism | protein RecA |
| TIGR00263 | *trpB* | Amino acid biosynthesis | tryptophan synthase, beta subunit |
| TIGR03632 | *uS11_bact* | Protein synthesis | ribosomal protein uS11 |
| TIGR00065 | *ftsZ* | Cellular processes | cell division protein FtsZ |
| TIGR01067 | *rplN_bact* | Protein synthesis | ribosomal protein uL14 |
| TIGR01021 | *rpsE_bact* | Protein synthesis | ribosomal protein uS5 |
| TIGR01049 | *rpsJ_bact* | Protein synthesis | ribosomal protein uS10 |
| TIGR00981 | *rpsL_bact* | Protein synthesis | ribosomal protein uS12 |
| TIGR01050 | *rpsS_bact* | Protein synthesis | ribosomal protein uS19 |

**Table S2.** List of genes involved in Kendomycin biosynthesis present in Streptomyces sps. VITGV156.

| **S. No** | **Identifiers** | **Position** | **Product** | **Homologous to** |
| --- | --- | --- | --- | --- |
| 1 | Ctg19.1 | 1 - 2737 | Type I polyketide synthase | UHY14125.1 *(kmy10)* *(S. verrucosispora)*;  CAQ52622.1 *(ken12) (S.violaceoruber)* |
| 2 | Ctg19.2 | 2744 - 7610 | Type I polyketide synthase | UHY14126.1 *(kmy11)* *(S. verrucosispora);* CAQ52623.1 *(ken13) (S.violaceoruber)* |
| 3 | Ctg19.3 | 7770 – 18426 | Type I polyketide synthase | UHY14127.1 *(kmy12)* *(S. verrucosispora);* CAQ52624.1 *(ken14) (S.violaceoruber)* |
| 4 | Ctg19.4 | 18427 - 28909 | Type I polyketide synthase | UHY14129.1 *(kmy19)* *(S. verrucosispora)* |
| 5 | Ctg19.5 | 28910 - 30020 | Type I polyketide synthase | UHY14130.1 *(kmy20)* *(S. verrucosispora)* CAQ52626.1 *(ken16) (S.violaceoruber)* |

**Table S3.** ADMET analysis of VITGV156

| **S.No** | **Chemical compound** | **Lipinski’s rule of 5** | **Molecular weight** | **Log P** | **H- bond acceptor** | **H- bond donor** | **Rotatable bonds** |
| --- | --- | --- | --- | --- | --- | --- | --- |
| 1 | Vicenistatin | Yes; 1 violation | 500.71 | 4.41 | 5 | 3 | 3 |
| 2 | Stambomycin | No; 3 violations | 1375.93 | 3.056 | 23 | 17 | 8 |
| 3 | Streptovaricin | No; 3 violations | 769.33 | 2.303 | 15 | 7 | 4 |
| 4 | Kinamycin | Yes; 1 violation | 454.1 | 2.689 | 11 | 4 | 4 |
| 5 | Kendomycin B | Yes; 0 violation | 470.64 | 4.26 | 5 | 2 | 0 |
| 6 | Methylenomycin A | Yes; 0 violation | 182.17 | 1.20 | 4 | 1 | 1 |
| 7 | Prejadomycin/ rabelomycin/  gaudimycin C | Yes; 0 violation | 324.33 | 2.13 | 5 | 3 | 0 |
| 8 | Granaticin | Yes; 1 violation | 444.11 | 3.128 | 10 | 7 | 0 |
| 9 | Versipelostatin | No; 3 violations | 1098.65 | 5.098 | 17 | 5 | 10 |
| 10 | Istamycin | Yes; 1 violation | 389.26 | -2.481 | 10 | 8 | 8 |
